# Modelos Preditivos para Aprimorar nosso Conhecimento: É Necessário?

**DOI:** 10.36660/abc.20210733

**Published:** 2021-10-06

**Authors:** Marcia M. Noya-Rabelo, Diogo Freitas Cardoso de Azevedo, Olga Ferreira de Souza

**Affiliations:** 1 Instituto D’Or de Pesquisa e Ensino SalvadorBA Brazil Instituto D’Or de Pesquisa e Ensino (IDOR), Salvador, BA - Brazil; 2 Hospital Aliança SalvadorBA Brazil Hospital Aliança, Salvador, BA - Brazil; 3 Escola Bahiana de Medicina e Saúde Pública SalvadorBA Brazil Escola Bahiana de Medicina e Saúde Pública, Salvador, BA - Brazil

**Keywords:** Doenças Cardiovasculares, Medição de Risco, Insuficiência Cardíaca, Hospitalização, China/epidemiologia

A insuficiência cardíaca (IC) é uma condição com prognóstico adverso; as taxas de mortalidade em 1 ano em estudos de base populacional foram relatadas em 35% a 40%.^1 - 5^ No Brasil, o registro BREATHE, grande amostra de pacientes hospitalizados com IC descompensada de diferentes regiões, demonstrou alta mortalidade intra-hospitalar (12,6%).^6^ Embora a internação por IC tenha diminuído de 2008 a 2017, foi observado aumento da taxa de tempo de internação por IC em pacientes hospitalizados.^7^ Informações prognósticas precisas podem aumentar nossa capacidade de prever resultados, informando assim as decisões aos pacientes. Inúmeros dados demográficos, variáveis clínicas e bioquímicas foram relatados para fornecer importante valor prognostico em pacientes com insuficiência cardíaca, e vários modelos preditivos foram desenvolvidos.^8^ Portanto, o conhecimento dos preditores de mortalidade pode ser usado para gerar modelos preditivos que podem auxiliar na tomada de decisão dos médicos identificando pacientes com alto ou baixo risco de morte.

Embora a maioria dos modelos anteriores sejam abrangentes e bem validados, ainda existem algumas limitações. Primeiro, alguns modelos foram construídos no período anterior ao recente tratamento farmacológico orientado por diretrizes. Em segundo lugar, a maioria dos modelos anteriores visava prever a taxa de mortalidade de curto prazo. Além disso, a maioria foi construída usando população de países ocidentais. Como já sabemos, os fatores que predizem a mortalidade no ambiente comunitário podem diferir.^9^

Nesta edição do Jornal, Wan et al.,^10^ apresentam o resultado de 1.742 pacientes com insuficiência cardíaca incluídos no Estudo de Pequim de Pacientes com Insuficiência Cardíaca e de Construção de uma Rede de Manejo da Insuficiência Cardíaca de outubro de 2015 a outubro de 2017. Esses pacientes foram aleatoriamente atribuídos em dois grupos: derivação (882 pacientes) e de validação (860 pacientes). O objetivo foi identificar fatores de risco correlacionados com a mortalidade em um ano entre pacientes chineses e desenvolver um simples escore de risco. A variável desfecho foi a mortalidade por insuficiência cardíaca em um ano, definida como morte por todas as causas após internação por IC. As informações de mortalidade foram obtidas por entrevista por telefone. As taxas de mortalidade em um ano para as amostras de derivação e validação foram de 7,3% e 5,8%, respectivamente (p=0,22). Wan et al.,^10^ identificaram cinco variáveis associadas à mortalidade em um ano: idade, sexo feminino, IMC, diâmetro atrial esquerdo e classe NYHA > 3. A área sob a curva ROC dessas cinco variáveis foi de 0,789, com boa discriminação. As variáveis finais identificadas foram consistentes com os fatores de risco previamente identificados nas coortes ocidentais. Verificou-se que o sexo feminino e o IMC são fatores protetores, e a classificação da NYHA, idade e diâmetro atrial esquerdo são fatores relacionados à mortalidade por IC, como sabíamos com base no achado anterior da população ocidental.

Existem vários modelos prognósticos validados, cada um deles combinando diferentes variáveis, o que sugere como é difícil estimar riscos em pacientes com IC. No entanto, os esforços para desenvolver e melhorar tais modelos probabilísticos são justificados porque o risco de mortalidade hospitalar, mortalidade após alta e reinternação ainda são elevados apesar da evolução do tratamento específico.

O uso de modelos probabilísticos melhora a precisão e oferece uma gama de probabilidades contínuas, aproximando o pensamento médico à melhor forma de lidar com a incerteza. No entanto, as imitações do presente estudo devem ser reconhecidas. Em primeiro lugar, a baixa incidência de mortalidade em um ano em pacientes com IC, embora 42% dos pacientes sejam da classe NYHA > 3. Segundo, algumas variáveis, que poderiam contribuir para o desfecho, não estão incluídas no modelo, como o tratamento farmacológico. Em terceiro lugar, a heterogeneidade na qualidade do atendimento entre os hospitais não foi medida. A associação entre qualidade hospitalar e mortalidade tem sido relatada em estudos anteriores fora e dentro da China.^11^ Outra possível limitação é a classificação incorreta de comorbidades com base na subnotificação no prontuário médico porque as condições coexistentes, mas não diagnosticadas, podem não ser reconhecidas ou ter variabilidade na abstração. Finalmente, o histórico médico da doença, especialmente doenças não circulatórias, pode ser relatado e documentado de forma incompleta. No entanto, isso resultaria em efeitos reais maiores porque tenderia a desviar os resultados em direção ao nulo. Além disso, existem limitações para a inferência causal sobre a relação entre qualidade e resultados devido à natureza observacional dos dados. No entanto, os resultados são congruentes com outros estudos publicados. O escore de risco visa identificar parâmetros independentes associados ao risco de mortalidade em vez de determinar a relação causal com a mortalidade. Neste escore, a maioria dos parâmetros significativos não são modificáveis. Portanto, este sistema de pontuação visa ajudar os médicos a prestar mais atenção aos pacientes com alto risco de mortalidade durante e após a hospitalização.

Os médicos geralmente avaliam a probabilidade de doença por um julgamento clínico não estruturado que pode subestimar ou superestimar o prognóstico em pacientes com insuficiência cardíaca com base em experiências clínicas passadas. Embora os médicos geralmente confiem nesse tipo de julgamento, ele tende a ser impreciso. Vários relatórios aludiram à necessidade de ferramentas para estimar o prognóstico quantitativamente. Em contraste com a experiência anedótica, o modelo preditivo de insuficiência cardíaca é uma estratificação precisa do risco de mortalidade. Esta escore simples, facilmente obtido e não dependente de um teste especializado, pode ser usado na China como uma estrutura para discutir o prognóstico e fornecer evidências para apoiar a tomada de decisão racional em pacientes com insuficiência cardíaca sob maior risco.
