# Supplementary material for: Modelos Preditivos para Aprimorar nosso Conhecimento: É Necessário?
Source: Arq Bras Cardiol. 2021 Oct 6;117(4):624–5. [Article in Portuguese] doi: 10.36660/abc.20210733 (PMC8528350; doi:10.36660/abc.20210733)
Supplement: Translation

# Translation: Predictive Models to Enhance our Knowledge: Is It Necessary?

**Authors:** Marcia M. Noya-Rabelo, Diogo Freitas Cardoso de Azevedo, Olga Ferreira de Souza

**Affiliations:** 1 Brazil Instituto D’Or de Pesquisa e Ensino (IDOR), Salvador, BA - Brazil; 2 Brazil Hospital Aliança, Salvador, BA - Brazil; 3 Brazil Escola Bahiana de Medicina e Saúde Pública, Salvador, BA - Brazil

**Keywords:** Cardiovascular Diseases, Risk Assessment, Heart Failure, Hospitalization, China/epidemiology

Heart Failure (HF) is a condition with an adverse prognosis; 1-year mortality rates in population-based studies have been reported to be 35% to 40%.^1 - 5^ In Brazil, BREATHE registry, large sample of hospitalized patients with decompensated heart failure from different regions, demonstrated high intrahospital mortality (12.6%).^6^ Although HF admission decreased from 2008 to 2017, it was observed increased rate of length of stay for heart failure in hospitalized patients.^7^ Accurate prognostic information may enhance our ability to predict outcomes, thus informing decisions for patients. Numerous demographics, clinical and biochemical variables have been reported to provide important prognostic value in patients with heart failure, and several predictive models have been developed.^8^ Therefore, knowledge of mortality predictors can be used to generate predictive models that can aid clinicians’ decision making by identifying patients who are at high or low risk of death.

Although most previous models are comprehensive and well-validated, there are still some limitations. First, some models were constructed in the period before the recent guideline-directed pharmacological treatment. Second, most previous models aimed to predict the short-term mortality rate. In addition, most were built using population in Western countries. As we have already known, factors that predict mortality in the community setting may differ.^9^

In this issue of the Journal, Wan et al.^10^ present the result of 1 742 heart failure patients included in the Beijing Monitoring Heart Failure Patients and Building Heart Failure Management Network Study from October 2015 to October 2017. These patients were randomly assigned into two groups: derivation (882 patients) and validation sample groups (860 patients). The aim was to identify risk factors correlated with one-year mortality among Chinese patients and develop a simple risk score. The outcome variable was one-year heart failure mortality, defined as all-cause death after an index HF hospitalization. The mortality information was obtained by phone interview. One-year mortality rates for the derivation and validation samples were 7.3% and 5.8%, respectively (p=0.22). Wan et al.^10^ identified five variables associated with one-year mortality: age, female gender, BMI, left atrial diameter and NYHA class > 3. The area under the ROC curve of these five variables was 0.789, with good discrimination. The final variables identified were consistent with the risk factors previously identified in Western cohorts. It was found that female gender and BMI are protective factors, and NYHA classification, age and left atrial diameter are factors related to HF mortality, as we knew based on the Western population’s previous finding.

There are several validated prognostic models, each of which combining different variables, which suggests how difficult it is to estimate risks in patients with HF. Nevertheless, the efforts to develop and improve such probabilistic models are justified because the risk of in-hospital mortality, mortality after discharge, and readmission are still elevated despite the evolution of specific treatment.

The use of such a probability model improves accuracy and offers a range of continuous probabilities, approximating medical thinking to the best form of dealing with uncertainty. However, the limitations of the present study should be recognized. First, the a low incidence of one-year mortality in HF patients even though 42% of patients are NYHA class > 3. Second, some variables, which could contribute to the outcome, were not included in the model, such as pharmacological treatment. Third, heterogeneity in the quality of care among hospitals was not measured. The association between hospital-level quality of care and mortality has been reported in previous studies outside and inside China.^11^ Another possible limitation is misclassification of comorbid conditions based on underreporting in the medical record because coexisting but undiagnosed conditions may not be recognized or have variability in abstraction. Finally, the medical history of the disease, especially non-circulatory illnesses, might be incompletely reported and documented. However, this would result in actual effects that are larger because it would tend to bias results toward the null. In addition, there are limitations to causal inference about the relationship between quality and outcomes because of the observational nature of data. However, the results are congruent with other published studies. This scoring system development aimed to identify independent parameters associated with mortality risk rather than determining the causal relationship with mortality. In this scoring system, most of the significant parameters are not modifiable. Therefore, this scoring system aims to help clinicians pay more attention to patients at high risk of mortality during and after hospitalization.

Physicians commonly assess the probability of disease by an unstructured clinical judgment which may underestimate or overestimate prognosis in heart failure patients based on passed clinical experiences. Although medical doctors usually put confidence into this type of judgment, it tends to be inaccurate. Several reports have alluded to the need for tools to estimate prognosis quantitively. In contrast to anecdotal experience, the heart failure predictive model is an accurate stratification of mortality risk. This simple score, easily obtained and not dependent on a specialized test, could be used as a framework to discuss prognosis and provide evidence to support rational decision-making in heart failure patients at the highest risk in China.
